# A Complex Obstetric Dilemma: Cesarean Scar Dehiscence

**DOI:** 10.7759/cureus.65373

**Published:** 2024-07-25

**Authors:** Mohini Gokuldas, Shriraj Katakdhond

**Affiliations:** 1 Obstetrics and Gynaecology, Dr. D. Y. Patil Medical College, Hospital and Research Centre, Dr. D. Y. Patil Vidyapeeth, Pune, IND

**Keywords:** scar thickness, previous caesarean section, scar tenderness, fetal distress, uterine scar dehiscence

## Abstract

In clinical practice, scar dehiscence following a previous cesarean section is a serious worry that necessitates close consideration of a number of contributing factors. We present the case of a 29-year-old gravida six, para three, who presented at 36 weeks of gestation with scar tenderness and abdominal discomfort at the site of her previous cesarean section scar. Despite a clear cardiovascular and respiratory examination, the lower-segment scar was notably thin at 1.2 mm, raising concerns for scar rupture. An emergency lower-segment cesarean section revealed a 4 x 2 cm scar dehiscence. The patient was counseled on the risks of future pregnancies and advised to consider tubal ligation. Early complications of cesarean delivery include wound hematoma, infection, and cesarean scar dehiscence (CSD), while long-term issues involve morbid adherent placentae and intra-abdominal adhesions. Short inter-pregnancy intervals and multiple cesarean deliveries are significant risk factors for CSD due to inadequate myometrial healing. Diagnostic imaging, particularly ultrasonography, is crucial for monitoring scar thickness and planning the timing of delivery. Management may involve conservative resuturing or hysterectomy in cases of severe infection or abscess formation. Early detection through vigilant prenatal care and monitoring, coupled with a multidisciplinary approach, can optimize maternal and fetal outcomes. Enhanced education for healthcare providers and expectant mothers, along with technological advancements, are key to improving the management of this complex obstetric dilemma.

## Introduction

The most prevalent uterine surgical procedure for women of reproductive age is a cesarean section, and globally, its prevalence has been continuously rising [1]. Uterine scar dehiscence or cesarean scar dehiscence (CSD) is an uncommon disorder that affects roughly 0.6% of cases globally. Many factors can lead to it, including previous lower-segment cesarean section, uterine trauma, congenital defects, irregular placentation, and improper oxytocin delivery. Patients may exhibit symptoms of endomyometritis, peritonitis, and postpartum hemorrhage shortly after giving birth or a few weeks later [2]. The reported incidence of CSD ranges from 0.2% to 4.3%. Uterine rupture during trial of labor (TOL) in a second pregnancy is known to be associated with prior CSD, but the clinical relevance of CSD involving recurrent cesarean sections is still unknown. Also, there is no recognized screening protocol for these instances, nor any recommended surgical technique for fixing CSD during delivery [3]. After lower-segment cesarean section, uterine dehiscence is a severe but uncommon complication that is frequently associated with the emergence of puerperal sepsis. Because of the impaired uterine closure and scar tissue integrity, this condition offers considerable risks, including the potential for infection-related consequences like peritonitis and abscess formation [2].

CSD which occurs in 8.3% of women with a history of prior cesarean section has a high risk and, if left untreated, can have devastating consequences. The risk is increased greatly by factors such as scar thickness, discomfort in the scar following surgery, and a brief inter-pregnancy period (less than 12 months). Scar dehiscence is also more common in women who have had three or more pregnancies, which highlights the need for close observation and spaced pregnancies for best results [4]. These risk factors highlight how difficult and crucial it is to recognize and keep an eye on possible problems with scar dehiscence in expectant mothers who have had cesarean sections in the past to guarantee the best possible outcomes for both the mother and the fetus.

## Case presentation

A 29-year-old female with a gravida of six, parity of three, living three, abortion of two, and nine months of amenorrhea arrived at the labor room with a 24-hour history of dull, throbbing abdominal discomfort at the site of the cesarean section scar. The pain was non-radiating. Her obstetric history revealed three live children delivered by lower-segment cesarean section and a pregnancy of 36 weeks and three days on the calendar. The single live intrauterine gestation at 35.4 weeks with an estimated fetal weight of 2.85 kg was seen on the most recent ultrasound performed during the ninth month of gestation. The lower-segment scar was 1.2 mm thick, and the placenta was anterior.

The patient had no other past medical or surgical history. Vital signs on admission included a blood pressure of 100/60 mm of mercury, a pulse rate of 120 beats per minute, and clear cardiovascular and respiratory sounds. On abdominal examination, the uterus was consistent with full-term pregnancy with fetal heart sounds regular at 120 bpm, with 1-2 contractions lasting for 5-10 seconds in 10 minutes, scar tenderness present, per vaginal findings with cervical internal os closed.

**Figure 1:**
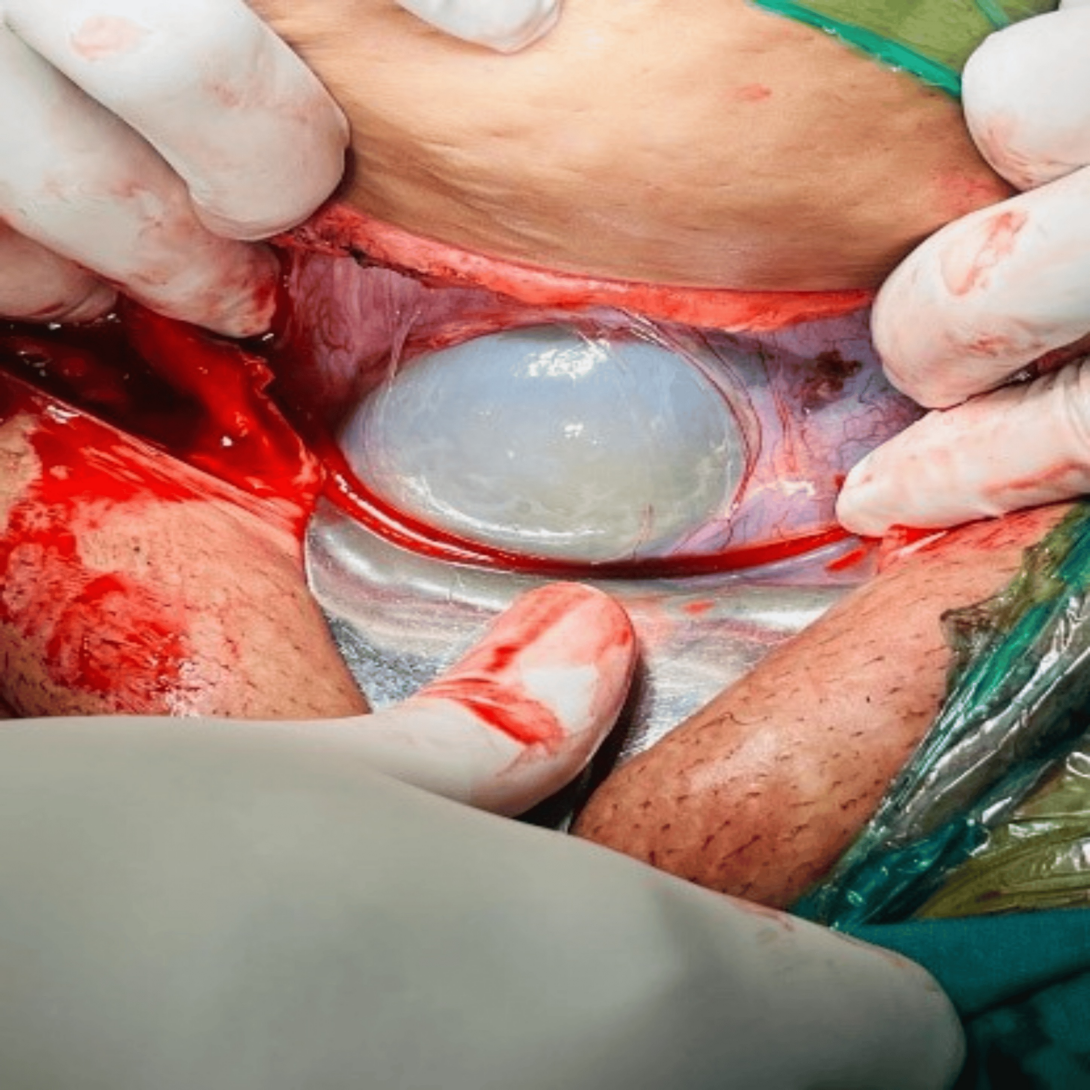
Intraoperative finding of scar dehiscence of size 4 * 2 cm

Emergency lower-segment cesarean section was performed due to scar tenderness and concern for scar rupture. She delivered a male weighing 2.8 kg with an intraoperative finding of scar dehiscence of size 4x2 cm (Figure 1).

The patient and relatives were counseled on the risks and benefits associated with further pregnancies and were advised for tubal ligation.

## Discussion

Early complications during cesarean delivery include wound hematoma, infection, and CSD; long-term complications include morbid adherent placentae and intra-abdominal adhesions with subsequent years of infertility [3]. Short inter-pregnancy intervals (less than 12 months) and prior cesarean sections performed before term are linked to CSD as it prevents the myometrium from having enough time to repair and strengthen. Because of this, the repaired myometrium is thinner and less able to withstand uterine distension. Pregnancy and labor increase the chance of dehiscence at the location of the previous incision [1]. Myometrial thinning is defined in various ways; however, it is not consistently defined when it comes to uterine incisions. Myometrial thinning is estimated to be caused by CSD and is seen in 0.2%-4.3% of post-cesarean pregnancies [3]. Myometrial thinning that occurs unexpectedly and extensively is one of the risks associated with cesarean sections, and it can be very challenging to treat. Wounds to the lower uterine section can expand transversely or downward, even though the fetus can be delivered. Additionally, there is a greater chance of damage to the bladder and other nearby organs [5].

Uterine dehiscence is primarily associated with the frequency of previous cesarean deliveries. If left untreated, dehiscence can cause symptoms that are not exclusive to pregnancy and may need to be corrected. Before further pregnancies, dehiscence should also be treated. Planned delivery before the commencement of labor, combined with diligent symptom monitoring, is the optimal management approach. Furthermore, in women who have had previous cesarean deliveries, ultrasonography should be utilized to evaluate the thickness of the lower uterine region [2].

Similar to transvaginal/abdominal ultrasound, the CT scan is still one of the greatest diagnostic instruments. The essential procedure for the diagnosis, management, and correction of uterine scar dehiscence is exploratory laparotomy. Conservative resuturing following debridement is an option; however, a hysterectomy should be taken into consideration if there is a significant wound infection, endomyometritis, or an intra-abdominal abscess [6].

## Conclusions

CSD is a rare but possibly fatal occurrence that necessitates thorough knowledge and prompt treatment. A thorough understanding of the symptoms and indications may help in early detection and prompt interventions. Management of such conditions requires prenatal care, identification of high-risk individuals, and vigilant monitoring during labor. The incidence and overall maternal and fetal outcomes can be improved with technological developments in medicine and improved education for both healthcare workers and expectant mothers. The best outcome for the mother and the baby can be ensured by a multidisciplinary strategy comprising obstetricians, anesthesiologists, neonatologists, and other healthcare specialists for the timely and efficient management of CSD.
